# Successful bilateral ureteral occlusion using liquid embolization for anastomotic insufficiency and insufficiency of an ileal conduit

**DOI:** 10.1055/a-2667-7088

**Published:** 2025-09-08

**Authors:** Julius Henry Loeser, Irem Bayram, Markus Wallwiener, Jörg Kleeff, Georgios Gakis, Florian Schanz, Walter A Wohlgemuth

**Affiliations:** 1 14942 Radiology, University Hospital Halle, Halle (Saale), Germany; 2 14942 Gynecology, University Hospital Halle, Halle (Saale), Germany; 3 14942 Visceral, Vascular and Endocrine Surgery, University Hospital Halle, Halle (Saale), Germany; 4 14942 Urology, University Hospital Halle, Halle (Saale), Germany

## Background

Rectovesical fistulas pose a surgical challenge in cases of high-grade/infiltrative endometrial carcinoma or bladder carcinoma and may require construction of an ileal conduit. This situation often results in an extended, complicated postoperative course with, for example, recurring pyelonephritis, wound-healing disorders, and urine leakage into the subcutaneous fatty tissue or the abdominal cavity 1 . Interventional occlusion of the ureter, for example, by gluing is an established treatment method, especially when surgical options are limited, or the conditions and postoperative wound healing are considerably impaired 1 2 3 4 .

Lava Liquid Embolic System used in the case presented here is a liquid embolizing agent for the intravascular treatment of peripheral vascular hemorrhage and has already been shown to be effective in embolizing arteriovenous malformations 5 .

## Clinical history

A 72-year-old female patient was admitted to the radiology department from the urology department of our clinic for bilateral anterograde ureteral occlusion. She had been diagnosed with a highly differentiated (G1) endometrioid adenocarcinoma and at that time treated externally solely with primary radiotherapy.

Ten years later, tumor recurrence, now with intracavitary spread, was detected during a gynecological check-up; thus, hysterectomy and bilateral salpingo-oophorectomy were performed externally. Histopathological analysis revealed a pT1a G2/3 tumor stage; no further treatment was initiated. Three years later once again a tumor recurrence was discovered, this time showing infiltration of the fundus of the bladder; thus, an external transurethral bladder resection was performed. During that time, a fistula of the vaginal stump to a loop of the sigmoid colon with involvement of the lumen of the urinary bladder was suspected for the first time.

The patient was transferred to the Radiology Department when her symptoms re-emerged: She was presenting with recurrent, irregular stools from the vagina as well as with recurrent pyelonephritis. A CT scan confirmed the suspected rectovaginal fistula and also showed severe recurrence of the previous endometrial carcinoma, now with para-aortic lymph node metastasis and infiltration of the bladder and both ureters, whereupon an interdisciplinary decision was made to first explore the pelvis and then perform ureterolysis on both sides and anterior and posterior exenteration with construction of an ileal conduit. Postoperatively, complications developed, including secondary bleeding, recurrent pyelonephritis, and insufficiency of the ileum conduit with subcutaneous tunneling and avulsion of the conduit from its anchorage; thus, surgical revision was carried out, but a definitive surgical repair was not possible. Even with external urinary drainage via Mono-J-catheters on both sides and percutaneous nephrostomy bilaterally, there was still an insufficiency of the ileal conduit with leakage of urine into the abdominal cavity. Because of difficult surgical conditions and a general palliative therapy regimen, further healing could no longer be anticipated. Therefore, the patient was referred to the radiology department for interventional bilateral anterograde ureteral occlusion.

## Intervention

**Fig. 1:**
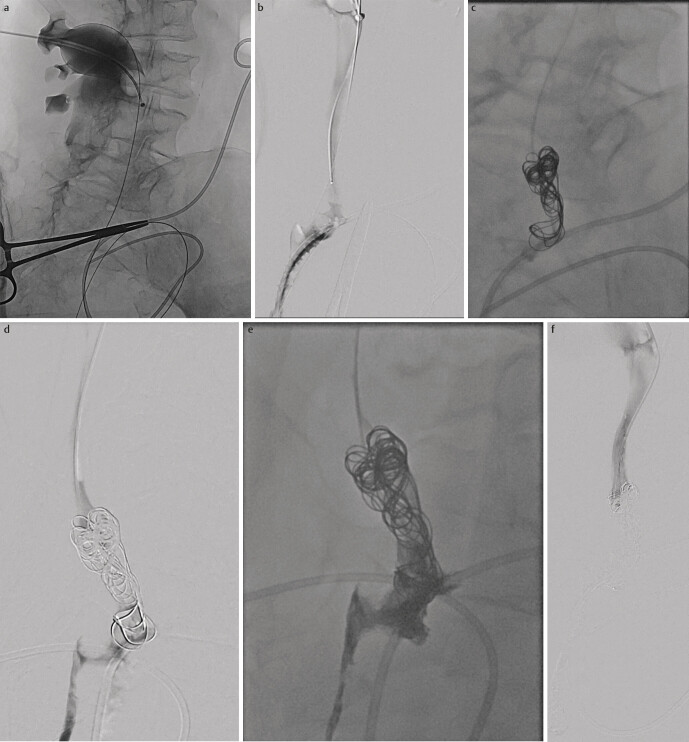
**a** : Contrast agent is injected into the right ureter via the percutaneous nephrostomy to visualize the current situation. **b** : In the area of the ureteral anastomosis, the contrast agent runs through the anastomotic insufficiency directly next to the ileum conduit situation. **c** : Several coils were released into each other in the distal ureter as a framework for the subsequent gluing. **d** : Under roadmap function, the glue can be seen passing through the coil bundle and continuing to flow into the abdominal cavity. **e** : You can see the ureter filled and occluded with ebolisate, with the coils contained. **f** : After repeated injection of contrast agent via the percutaneous nephrostomy, the distal ureter can be seen occluded under the Roadmap function and without leakage into the abdominal cavity.

The anterograde occlusion of both ureters was performed in two sessions under general anesthesia. During the first intervention, contrast agent was injected into the right-sided percutaneous nephrostomy under fluoroscopy for orientation and to evaluate the situation. Through a microcatheter, which was inserted into the right ureter via the percutaneous nephrostomy, it was possible to display the course of the ureter and visualize an anastomotic insufficiency of the distal part with direct contrast agent outflow into the abdominal cavity from the right side of the ileal conduit. Using different sizes of coils (20 mm, 16 mm, and 14 mm) successively released one into another using a suitable roadmap, a framework for the planned gluing could be established. After the microcatheter was moved forward to the distal part of the anastomosis area of both ureters (Wallaceʼs ureteral plate), we initially administered a small amount (2 ml) of the 1:1 mixture of Magic glue and Lipiodol used for gluing under fluoroscopy. However, the glue was too liquid and was able to pass directly through the anastomotic insufficiency without showing any adhesive effect. Instead, therefore, Lava Liquid Embolic System was used to continue the intervention and occlude the ureters: Lava has already been used successfully as an embolic agent in the treatment of peripheral vasculature hemorrhage and arteriovenous malformations. With a total of 3 ml Lava 34, we were able to control any expansion and avoid reflux along the catheter by gradually injecting the embolizing agent and waiting about 60–90 s between injections for the increasing polymerization-related solidification of the agent. In this way, the right distal ureter was successfully occluded, including the coil bundle for structural stabilization. In a final control series using anterograde administration of contrast agent, the right ureter did not show any leakage ( Fig. 1 ).

**Fig. 2:**
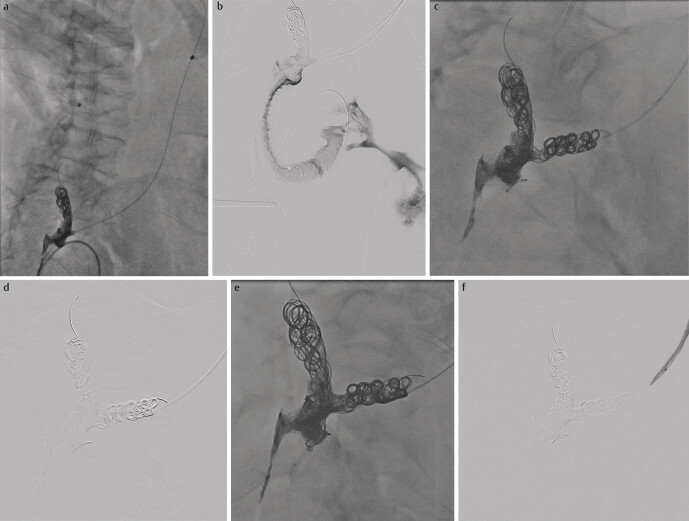
**a** : A microcatheter was used to access the left ureter while the package of coils and embolisate on the right side is shown. **b** : It can be seen that the upper anastomosis of the ureters was occluded by the first intervention, but contrast agent flows into the abdominal cavity through an insufficiency of the ileal conduit. **c** : Multiple coils were released into each other in the left ureter distally in the area of the Wallace plate. **d** : The gradual filling of the coil framework with embolisate can be seen increasing from proximal to distal. **e** : Most of the coil framework was filled with attachment to the package from the first session while avoiding reflux along the catheter. **f** : Contrast agent was administered again via the left percutaneous nephrostomy, with neither leakage of contrast agent through the packages of coils and embolisate nor leakage into the abdominal cavity being visible.

Following the interventional occlusion of the right ureter no complications developed and therefore the left ureter was approached in a second session. As on the opposite side, the ureter was similarly approached and visualized via the left-sided percutaneous nephrostomy using a microcatheter. No insufficiency was detected at the upper anastomosis of the two ureters; therefore, we assumed that this side was also occluded as a result of the first intervention. However, a wide-open leakage from the ileum conduit into the abdominal cavity was found far distally. Again, the microcatheter was moved forward to the anastomosis area of the ureters and after building up a framework of coils (16 mm, 12 mm), the anastomosis area, and therefore both ureters, could now be completely closed by gradually injecting a total of 2 ml Lava. In the final control series also using anterograde injection of contrast agent, the left ureter also appeared to be occluded in the distal area ( Fig. 2 ).

## Follow-up

The ureters and the associated insufficiencies were closed in separate sessions. No complications were observed after the first use of the Lava Liquid Embolic System in the urinary tract, also in terms of a short-term follow-up between the two interventions. After the second intervention, no complications associated with the ureteral occlusion were detected either. As no complications developed during the postinterventional course, the patient could be transferred to a rehabilitation facility after a short stay in the hospital. The symptoms previously caused by urine leakage were decreasing.

## Discussion

In the patient presented here, we were able to completely occlude both ureters, resolve the anastomotic insufficiency in the presence of a rectovesical fistula, and create an ileal conduit in a recurring, highly metastatic, and infiltrative endometrial carcinoma using a coil pack and Lava Liquid Embolic System. This system offers not only an alternative to interventional ureteral occlusion when surgical options are limited, but also a new option when previous gluing of the ureter fails. Moreover, this system also represents an alternative to laparotomy, particularly in difficult surgical circumstances, for example, obesity per magna, with access via a percutaneous nephrostomy and following an anterograde approach 3 4 .

The quick recovery time after the intervention, the absence of larger wounds due to surgical incision, and the resulting absence of any risk of wound infection or wound-healing disorders can also be considered beneficial aspects. However, this procedure is technically challenging and requires both reliable and safe handling of catheters that are otherwise only used in the vascular system and expertise in handling liquid embolic agents; therefore, these procedures are mainly performed at specialized centers. However, considering the severe and recurrent findings in this patient, treatment at a specialized center with contributions from several medical specialties seems reasonable and effectively demonstrates the value of interdisciplinary teamwork.

When performing embolization therapy, special attention must also be paid to a possible reflux of the embolizing agent alongside the catheter in order to prevent the tip of the catheter from getting stuck; therefore, the embolizing agent was only injected gradually. Furthermore, this method also prevents the embolization material from escaping into an unintended area. Therefore, special attention must be paid to these two points when using liquid embolizing agents, filling the area in a way that is adapted to the local structural characteristics and thereby reducing the probability of a secondary or undetected leak.

In conclusion, we have shown that interventional occlusion of the ureters using coils and the Lava Liquid Embolic System is a safe and effective, though technically challenging method of occluding the ureters in cases of insufficiencies. However, further studies are needed to confirm these findings.
